# Biased perceptions of public opinion don’t define echo chambers but reveal systematic differences in political awareness

**DOI:** 10.1371/journal.pone.0324507

**Published:** 2025-06-04

**Authors:** Crispin H. V. Cooper, Kevin Fahey, Regan Jones

**Affiliations:** 1 Sustainable Places Research Institute, Cardiff University, Cardiff, United Kingdom; 2 School of Geography and Planning, Cardiff University, Cardiff, United Kingdom; 3 School of Computer Science and Informatics, Cardiff University, Cardiff, United Kingdom; 4 School of Politics and International Relations, University of Nottingham, Nottingham, United Kingdom; National Taiwan University, TAIWAN

## Abstract

Echo chambers are widely acknowledged as a feature of online discourse and current politics: a phenomenon arising when people selectively engage with like-minded others and are shielded from opposing ideas. Various studies have operationalized the concept through studying opinions, interactions, reinforcement or group identity. Echo chambers both feed and are fed by the false consensus effect, whereby people overestimate the degree to which others share their views, with algorithmic filtering of social media also a contributing factor. Although there is strong evidence that meta-opinions - that is, people’s perceptions of others’ opinions - often fail to reflect reality, no attempt has been made to explore the space of meta-opinions, or detect echo chambers within this space. We created a new, information-theoretic method for directly quantifying the information content of meta-opinions, allowing detailed exploratory analysis of their relationships with demographic factors and underlying opinions. In a gamified survey (presented as a quiz) of 476 UK respondents, we found both the liberal left, and also people at both extremes of the left/right scale, to have more accurate knowledge of others’ opinions. Surprisingly however, we found that meta-opinions, although displaying significant false consensus effects, were not divided into any strong clusters representative of echo chambers. We suggest that the metaphor of discrete echo chambers may be inappropriate for meta-opinions: while measures of meta-opinion accuracy and its influences can reveal echo chamber characteristics where other metrics confirm their presence, the presence or absence of meta-opinion clusters is not itself sufficient to define an echo chamber. We publish both data and analysis code as supplementary material.

## Introduction

Echo chambers are widely acknowledged as a feature of online discourse and current politics; the “trenches in which culture wars are fought” by opposing tribes [1] as people follow elites sharing their partisan predisposition [2] via selective media consumption [3,4] and network effects [5–7], leading to increased political polarization and ultimately threatening the democratic process itself [8–11] including management of the climate crisis [12,13].

Terren and Borge-Bravo [14] systematically reviewed 55 studies of echo chambers, with a definition based on the assumption that “social media users selectively engage with like-minded others and ideologically-aligned content, thus rarely being exposed to the conflicting ideas that make up the agonistic public sphere”. A more recent systematic review by Mahmoudi et al. [15] covered 28 studies and notes that there is not yet a universally accepted, formal definition of an echo chamber; however, studies can be classified based on their use of one or more of the following components of a definition: (1) the formation of a group by like-minded individuals, (2) the prevalence of interactions within the group compared to interactions with users outside the group, and (3) the reinforcement of beliefs within the group. These are termed, respectively, the semantic, structure, and reinforcement components of the echo chamber. Other literature focuses instead on group identity-building aspects [16,17]: Cinelli et al. [17] makes the link to group polarization theory [10], stating that echo chambers can move entire groups towards more extreme positions. Related work examines how these dynamics are weaponized in the political arena, including to spread disinformation [1,18]. Notwithstanding, membership of an echo chamber may be rational from an individual perspective. Although it has long been recognized that many voters do not have consistent political beliefs [19], the framework of bounded rationality recognizes that beliefs must be constrained by access to information and processing power [20]. Within this context, echo chambers may form a useful heuristic for individuals to determine their own beliefs [21].

Based on the above definitions, various studies operationalize the echo chamber concept by measuring inter-personal online interactions [6–8,17,22,23]. Others measure media consumption [3,4,24,25], and a few measure both [26,27]. These studies have shown a variety of results, some strongly supporting the existence of echo chambers [3–7,17,22] while others suggest the phenomenon may not be so strong as generally believed [8,23–27].

The current study takes a different approach by focusing on perceptions of the views of others, or “what people think other people think”. Existing work terms these either second order beliefs [12] or meta-opinions [18]. Meta-opinions naturally form part of the echo chamber dynamic. Misperceptions of what others think can be self-reinforcing when people are reluctant to share opinions they believe differ from the majority [28]. This can lead to different outcomes for individuals depending on their privately held views: those whose views match publicly shared opinion experience false consensus, in which people overestimate the degree to which others share their views [29,30] whereas those whose views differ experience the opposite - in extreme cases leading to pluralistic ignorance at group level, in which the majority of the group misunderstand one another [31,32]. In cases where these effects are self-reinforcing they are also referred to as a “spiral of silence” [18]. Algorithmic filtering of social media can also contribute to false consensus [24,27]. Confirmation bias, in which people have a tendency to be less critical of information that agrees with their existing beliefs (and more critical of information which conflicts) [33] can also increase attitude polarization [34] leading to reduced diffusion of information in clustered networks ([35]; this reference does not refer to the effect as an echo chamber, but reduced diffusion of information between clusters could be understood as part of the same phenomenon). All of these aforementioned effects could potentially influence echo chamber dynamics, with different groups of people holding different meta-opinions shaped by the members of the group. Where echo chambers exist, this leads to the possibility that within each echo chamber – although privately held opinion may vary – publicly shared opinion, and hence meta-opinion, will be biased towards the perceived consensus of the individual echo chamber.

We therefore set out to measure whether echo chambers might be visible as clusters in meta-opinion data. Although recent work has revealed strong misperceptions of the opinions of others at population level [11,12,31], we are not aware of any attempts to directly detect echo chambers as clusterings in the space of meta-opinions.

The contribution of the current study is (1) to provide an exploratory analysis of the space of meta-opinions, and (2) to publish the meta-opinion data we collected in a UK study conducted as a gamified survey. It should be emphasized that we do not perform any formal hypothesis testing. Scheel et al [36] reviews the various purposes of non-confirmatory research of this nature, under which our work can be classed as development of a measurement technique, and descriptive observation of the association between variables.

We introduce and validate a new, information-theoretic method for directly quantifying the information content of meta-opinions. This allows us to measure accuracy of meta-opinions while correcting for which meta-opinions are easier or harder to guess, especially in cases where more than two responses to an opinion question are possible (e.g., the correct answer also includes a proportion of people who are undecided on an issue). This in turn allows us

(1) to structure the meta-opinion survey as a quiz, with participant scores for each question providing feedback that reflects their level of knowledge rather than the inherent difficulty of each question, and thus enabling participants to learn from the quiz,
(2) to conduct detailed exploratory analysis of the relationship between meta-opinions, underlying opinions and demographic factors with confidence that findings reflect the underlying social dynamics influencing each topic, rather than the differences in question difficulty inherent in the question structure.

Although we began the study with the expectation that echo chambers may be visible as clustering in the space of meta-opinions, this was not apparent in our data. We therefore suggest that while meta-opinions are strongly linked to echo chambers – and measures of meta-opinion accuracy and its influences can reveal social dynamics relevant to echo chambers, and measure echo chamber characteristics where other metrics confirm their existence – that absence of clustering of meta-opinions themselves doesn’t itself indicate absence of an echo chamber. The analogy of an ‘echo chamber’ may be inappropriate for meta-opinions, which form a continuous space characterised by a false consensus effect centred on each individual in isolation, rather than a discrete space comprising a chamber for each political tribe.

## Materials and methods

We surveyed 476 United Kingdom residents for their opinions on a selection of politicized topics including trust for public institutions, free speech, construction of new homes, environment, body image, gender and transgender issues, Brexit, and further questions defining a 3-d political compass with dimensions left/right, libertarian/authoritarianism, pro/anti welfare. Alongside their own opinions, respondents were asked to guess the proportion of the UK population holding each opinion according to previous British Social Attitudes surveys [37,38]. We also included standard demographic questions, a survey of media types and sources consumed, self-reported political interest and a brief test of factual political knowledge.

### Sample and recruitment

The sample was based on paid recruitment of adult (age 18+) UK participants via Amazon Mechanical Turk, between 8^th^ March and 12^th^ April 2021. The first page of the online survey explained the study and proposed usage of participant data, and required participants to click to consent or withdraw.

The sample includes representation of all major political parties, all levels of education and all possible responses to our opinion questions (descriptive statistics provided as supplemental data). As is recommended practice in use of Mechanical Turk [39–43], we incorporated attention checks to help ensure respondents were providing thoughtful answers. We also rejected responses completed in under 2 minutes, or in which response sliders for meta-opinions remained unmoved from their default positions on 13 or more of the 20 meta-opinion questions. Both of these thresholds were determined by assessing what we felt to be reasonable. As respondents might often believe that default slider positions (representing an even split of opinions) were as good as their own guess, we allowed this for a majority of questions, with rejections for this cause accounting for only 1.2% of responses. The time threshold was based on our own fastest test runs of the quiz, which professional survey-takers might be expected to match; rejections for excessive speed accounted for an outlying 2.6% of responses. We also launched survey batches at differing times of day, and did not limit the sampling to experienced respondents only.

Ethical review was carried out in accordance with the School Research Ethics Committee procedures of the Cardiff University School of Geography and Planning, 2020. These procedures allowed supervisor approval for projects using a common methodology, provided no issues were identified through a screening checklist (covering, e.g., vulnerable individuals, possible harm). The project used standard methods for online survey with anonymised responses, and no issues were identified during screening, therefore an individual review was not required. Project details were filed with the committee. Informed participant consent was obtained through online form at the start of each survey. The draft dataset for public release was also independently assessed for statistical disclosure in 2023 by the Cardiff University School of Computer Science & Informatics Research Ethics Committee, confirming that risk of individual identification is negligible.

### Gamification

Although results were collected in the context of a survey, with respondents made aware of the requirement to engage seriously with the task to receive payment, the meta-opinions section was structured as a quiz with game-like aspects. The interface consisted of either two or three sliders per question, allowing the respondent to specify the proportion of the population they believed to hold each opinion. Sliders were constrained to sum to 100% by implementation of the following interface behaviour:

1. Adjusting any polarized response slider (e.g., Agree, Disagree, Trust or Distrust) caused the opposite slider to move in the opposite direction.
2. Adjusting any neutral slider (e.g., “Neither agree nor disagree”) would move both other sliders in the opposite direction, while maintaining a constant ratio between the other two sliders.

Respondents were informed of their score on completion. They were permitted, but not required, to explore their results on a question by question basis: this was presented as a single page in reverse score order, i.e., giving most emphasis to questions where the respondent’s guess of UK meta-opinions was most wrong. Following this, and without prior warning, they would be re-tested on the two questions on which they had scored least well, to determine whether they had engaged with the results page. A final page asked for feedback on the survey, as a Likert scale with optional free-text comments.

### Definition of variables

The full list of variables, and the survey questions themselves, are provided as supplemental data. Media consumption variables were based on asking whether the respondent consumes any newspaper, online news, televised news or social media site, 3 or more times per week. Where ‘yes’ was answered to any of these questions, participants were asked to name which, as a free text response. Sources that were used by more than 10 respondents (2.1%) were encoded as binary variables. For textual news, online and print sources were combined. Political knowledge is assessed by responses to three factual questions.

Political opinion questions, along with the population-wide distribution of responses used for scoring the quiz, were derived from the most recent British Social Attitudes Surveys (BSAS) available at the time [37,38]. BSAS defines three attitude scales forming a political compass (Left-Right, Libertarian-Authoritarian, Welfarism). Our original desired question set contained all 19 questions contributing to the three attitude scales, plus a manual selection of further BSAS questions relevant to ongoing political debates, including some from earlier BSAS years, with responses weighted according to BSAS user guidance. The original set comprised 61 questions in total, however pilot survey rounds indicated that people were unlikely to produce meaningful guesses for all 61 questions. To reduce the cognitive load on participants we therefore reduced the set to 20 questions; 17 from the 2018 survey (including 3 for each axis of the political compass) and 3 from the 2017 survey which we wished to ask but were not included in the 2018 survey. Participants were informed of the year of data collection for each question when guessing responses. Likert scales were simplified by merging some response categories (for example combining “agree” with “strongly agree”) to produce a maximum of 3 possible answers to each quiz question.

Opinion and meta-opinion variables were recoded for analysis. For opinion variables, individual Likert responses are rescaled to the 0–1 range. For political compass variables, variable names reflect the scale ends, e.g., LeftRight implies Left = 0, Right = 1. Meta-opinions are recoded as two variables per question, (1) the proportion of people the respondent believed would be undecided (denoted in figures by “UNDEC”, where data available), and (2) the proportion of the remainder that the respondent believes would have an opinion closer to 1 than 0 on the recoded Likert scale (in all figures denoted by “pO1”). Alongside each meta-opinion we also encode respondent score for the same question to indicate, where meta-opinions differ, which group of respondents holds a more accurate picture of the population level distribution.

### Information theoretic scoring

Quantifying the accuracy of distributional guesses requires correction for the fact that it is easier to guess some distributions than others: for example, where the population is split 50%/50% between two opinions, then by chance alone, guesses are likely to be closer to the truth than for a split of 100%/0%. Correctly guessing a 100%/0%/0% split between three opinions would be harder still. Initial piloting of the meta-opinion quiz revealed that participants’ ‘most wrong’ answers often occurred due to a distribution which was inherently harder to guess for this reason, and not because the participant was misperceiving public opinion *per se*. We therefore developed an information-theoretic scoring system to correct for this effect.

The system scores individual responses by quantifying the bits of information each respondent got right, over and above what would be expected from a random guess. This has three advantages: (1) Standardized measurement of small differences in individual levels of knowledge, across easier and harder questions, enables exploratory analysis of the relationship between demographics, opinions and accuracy of meta-opinions with confidence that question-level effects relate to social factors surrounding each question rather than the numerical properties of the distribution of opinions. (2) Including three-option questions in this way also enables us to ask respondents to guess the proportion of population undecided on some issues (where data are available), which we would expect to be systematically underestimated in the echo chamber scenario. (3) Scoring which more accurately reflects the respondent’s degree of success on each question, is useful in feedback to participants which is an essential component of the gamification of the survey.

Information theory was first formalized in the 1940s by Shannon [44]. The key insight is that low probability events carry high information, and vice versa. If we observe an event that we knew was certain to happen already, then the observation of the event tells us nothing new; conversely if we observe an event that we thought to be highly unlikely then we have learned something. Information theory also formalizes our intuition that the information carried by two independent events A and B should simply be the information in A plus the information in B. Note that this is different to probabilities, where the probabilities of A and B must be multiplied (not added) to determine the probability of them both occurring. These facts together lead to defining the information content of an event as the negative log probability of its occurrence. Typically this is expressed as a logarithm with base 2, reflecting the fact that our computers store information in ‘bits’ which can take one of two states (0 or 1). For perfectly coded data, the information content in bits is exactly equivalent to the number of ones and zeros required to store it. Fractional bits of information arise because imperfectly encoded information can theoretically be compressed to a smaller average number of bits per event.

In the case of our scoring, participants make guesses of a given quality. The event of interest is the probability of a participant making a guess of equal or better quality by chance alone. We therefore define the information content of a guess g at a true distribution t, as the negative log probability of achieving a guess of at least equal quality by chance:


$$bits(g,t)=-\log_{2}\frac{\left|x\in G:quality(x,t)\geq quality(g,t)\right|}{\left|G\right|}$$ (1)

where G is the population of all possible guesses including the true distribution, i.e., for a distribution over n parameters taking discrete values in the range 0…r which must sum in total to r,


$$G=\left\{\;\left(x_{1},\ldots ,x_{n}\right)\;\right|\;x_{i}\in \left\{0,\ldots ,r\right\}\;,\;\Sigma_{i=1}^{n}x_{i}=r\;\}$$ (2)

We define *quality(g,t)* the quality of a guess g of truth t, as the negative sum of pairwise absolute differences between values,


quality(g,t)=−Σ |g−t| (3)

noting that the user interface presented to respondents automatically constrains slider positions to ensure that the vector *g* sums to 100%. The *correct* information content of a given guess is then defined as its information content over and above the mean information content of a random guess:


$$score(g,t)=bits(g,t)-\overset{―}{bits(x,t)}{|}_{\;x\in G}$$ (4)

### Clustering analysis

For clarity it is worth emphasizing that the clustering analysis in this paper is applied to meta-opinions themselves, and not social network data as with existing literature. It tests whether the respondents can be divided into well-defined groups based on their meta-opinions alone: for example given a set of possible opinions {A, B, C, …}, can you roughly describe the respondents as a small number of groups, where one group believes the majority think ABCDE, another group believes the majority think FGHIJ, and so on. To cluster in this manner it is not necessary that a group agrees unanimously on what others think; for example in the ABCDE group some may believe ABCD while others believe BDE or even - in moderation - include beliefs from other groups, for example BDEJ. What would matter overall is the extent to which this group is separable from other, obviously different groups such as FGHIJ.

To test for clustering we attempted application of: (1) the k-means clustering algorithm [45], tested for all values of n (the number of clusters) from 2 to N-1 (where N is the number of valid survey responses) and repeated with 100 random initializations of the means in each case; (2) the OPTICS algorithm [45,46]. We tested separation of clusters thus derived using the silhouette coefficient [47] which is defined for each sample *s* as


$$silhouette_{s}={(b}_{s}-a_{s})/max(a_{s},b_{s})$$ (5)

where $a_{s}$ is the mean distance between a sample s and all other points in the same class, $b_{s}$ is the mean distance between that sample and all other points in the next nearest cluster, and the overall cluster separation is reported as the mean silhouette score over all points in the dataset. Thus, a silhouette score of 0 indicates fully overlapping (i.e., meaningless) clusters, while a silhouette score of 1 indicates the strongest possible clustering.

### Statistical analysis

Analysis is conducted in the Anaconda Python ecosystem using SciPy 1.6.2 [48], statsmodels 0.12.2 [49], Pandas 1.2.3 [50,51] and Matplotlib 3.4.1 [52]. Code and data are provided in S3 File.

In the results section, we quantify association between variables using Pearson correlation. Reported p values give the probability of obtaining the measured correlations (or stronger ones) in our sample, under the null hypothesis that each pair of variables is uncorrelated at population level. It is worth emphasizing that – in the context of our non-confirmatory approach - such p values should not be mistaken for a formal test of any specific hypothesis; rather, they are used to provide a cursory indication of whether observed correlations may be spurious. To mitigate the well-known limitations of p values [53] we graphically present some results as regression coefficients with confidence intervals to make clear the distinction between effect size and significance. Where space does not permit this approach, the arbitrary threshold of p = 0.05 appears as only one of several divisions on a graded colour scale.

We also present correlation matrices which by their nature encompass large numbers of pairwise correlation tests, with the associated risk that spurious correlations will appear due to multiple testing (that is, if we expect 1 in 20 correlations to be spuriously significant but we test 1,000 correlations, we would expect to find 50 spuriously significant correlations which are purely due to chance in the sample, rather than revealing a true association between variables). Throughout the results section we therefore discuss the effect of mitigating this effect with Bonferroni correction of the relevant p values. Moran [54] argues that Bonferroni correction is overly conservative, i.e., increases the likelihood of false negative results to the point where true associations between variables are likely to be deemed insignificant. For the purpose of an exploratory study we find this limitation acceptable, leading to the following interpretation of our correlation matrices: (1) cells which remain significant after Bonferroni correction are likely to be of interest; (2) where cells do not remain significant after Bonferroni correction there is a risk, but not certainty, that they may represent spurious correlations.

### Filtering correlation matrices

To check for correlations unexplained by the false consensus effect (i.e., to allow inspection of the residuals of an exploratory false consensus model) we filter some correlation matrices. To achieve this, we note for each meta-opinion variable M, there is a corresponding opinion variable O which, under the false consensus hypothesis, we would expect to be positively correlated. We then consider each independent variable X. If the relationships between X and M, and the relationship between X and O, can both be modelled as monotonic functions then we expect the sign of correlation(X,O) to match the sign of correlation(X,M). In cases where these signs match, we therefore do not display the corresponding cell (X,M) in the filtered correlation matrices. We split the remaining correlations into two categories: (1) if correlation(X,O) is not significant at p = 0.05 then we classify the effect (X,M) as unexplained by, but not significantly contradicting false consensus; (2) if correlation(X,O) is significant at p = 0.05 then we classify the effect (X,M) as significantly contradicting false consensus. We do not display the squared independent variables (LibAuth2, LeftRight2, WelfProAnti2, OPIN_takes_sides_Brexit) in filtered correlation matrices as their relationships with M and O are not monotonic.

## Results

Scores of knowledge, quantified in bits of information, were computed for all instances of questions answered by all respondents. The best score for a single question answer was 10.9 bits, which corresponded to an exceptionally good guess differing from the truth by only 0.38%pt (percentage points, averaged over the three possible opinions for that question). The worst single score was -1.4 bits, representing a worse-than-random guess with error of 48%pt (the respondent had guessed an opinion split of 0/100/0% against a true split of 37/27/35%). A sample guess with performance close to random (score of zero bits) differed from truth by 19%pt.

Considering average scores per respondent, the top decile of respondents performed well, with information scores averaging 1.8–1.3 bits per question and average errors 10–13%pt. The median respondent averaged 0.6 bits per question with average error 18%pt. This is better than random: the respondent with a score closest to zero (approximating all random guesses) had average error of 23%pt. The worst performing respondent had guesses worse than random, averaging -1.0 bits with average error 35%pt.

To test the extent to which respondent error might be explained by respondents confusing public opinion during the study year (2021) with public opinion sampled for the quiz (2018), we checked differences in public opinion for questions which we were both used in our quiz, and repeated by BSAS in 2021 [55]. We found 10 such questions, with the mean difference between survey years for these questions being 5.5%pt.

We validated our approach by testing that that knowledge of others’ opinions is consistent for each respondent, i.e., someone scoring highly on one question is more likely to score a high average on other questions. The Pearson correlation of individual question scores for the same participant was r = 0.19, which was highly significant with p = 7e-75 (n = 9520).

**Fig 1:**
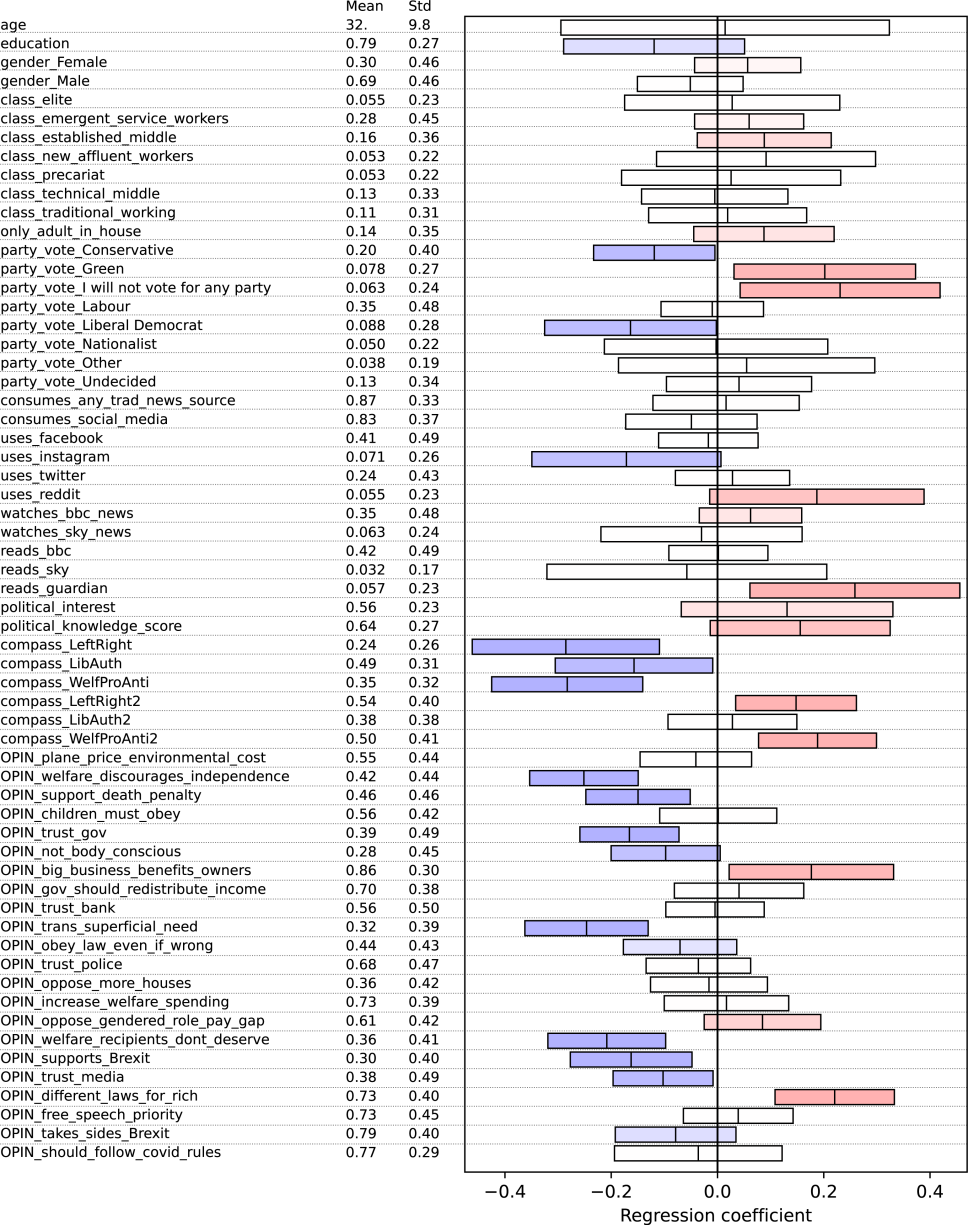
Descriptive statistics and bivariate regression coefficients between all independent variables and respondent score. Red shows positive correlation, blue shows negative. Independent variables are min-max scaled. Regression coefficients can be interpreted as total bits per question attributable to each independent variable. Bars show 95% confidence intervals; n = 476.

To determine which factors may most influence meta-opinion accuracy – that is, who has better knowledge of the opinions of others, versus who might inhabit a strong echo chamber – we performed bivariate regression of meta-opinion scores against each collected variable (Fig 1). The strongest coefficients (p < 0.05, n = 476, effect sizes up to 0.29 bits/question or 5.7 bits over the full survey) show knowledge of others’ opinions to be significantly higher for left/liberal/pro-welfare respondents as well as those with opinions on either extreme of the left/right and welfarism scales; those who voted for the Green party or refuse to vote for any party, those who view gender transitions as reflecting a significant or long-term need, Remainers, Guardian readers, and those who distrust government and media. Conversely, lower knowledge of others’ opinions was found for Conservative and Liberal Democrat voters. Weaker associations (0.05 < p < 0.08, effect sizes up to 0.19 bits/question) included a negative effect from Instagram users, a positive effect for Reddit, positive effect of political knowledge, negative effect for those less attached to their body image. It should be noted in interpreting these findings that although the sample used includes all major political parties, levels of education and responses to our opinion questions, it is self-selected rather than statistically representative.

**Fig 2:**
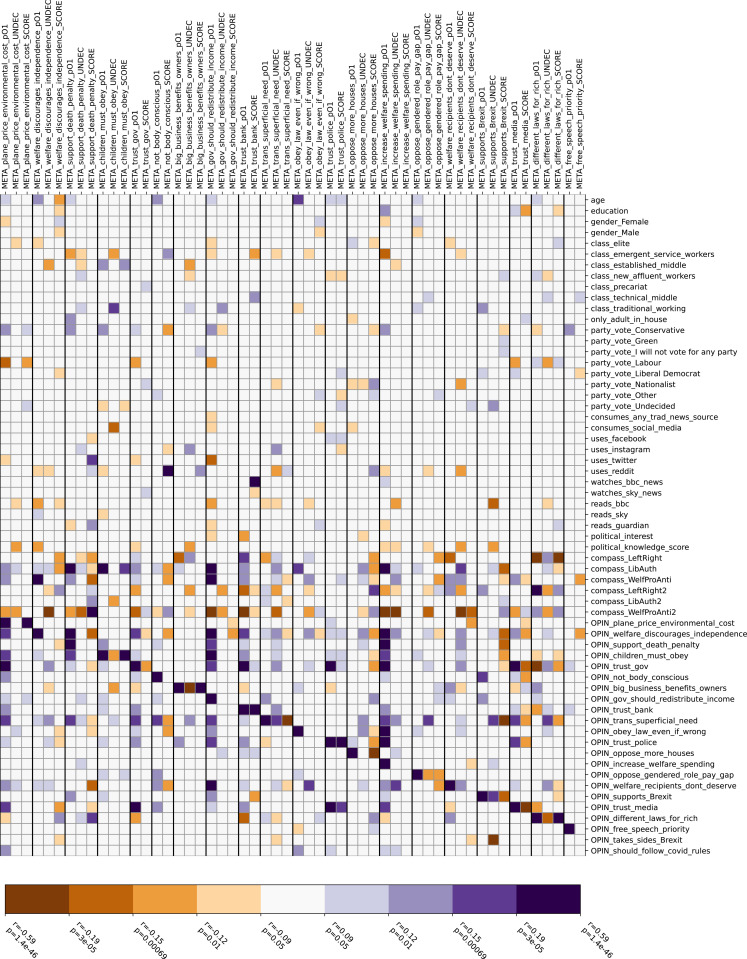
Correlation (Pearson r) of demographic and opinion variables with meta-opinion variables. Reds show negative correlation, blues shows positive. Note all colour bands in the legend are significant at p = 0.05. The strongest colour bands remain significant at p = 0.01 after Bonferroni correction. OPIN_ denotes respondent opinion for each question. META_ denotes meta-opinions. Suffix _UNDEC denotes proportion of population respondent believes would be neutral/undecided (corresponding to ordinal encoding of 0.5 in OPIN variable). Suffix _pO1 denotes proportion of population respondent believes chose option 1 (corresponding to ordinal encoding of 1 in OPIN variable). Suffix _SCORE denotes bits of information correctly guessed by respondent on each question.

To explore bias in the nature of meta-opinions, we tested for correlations of meta-opinions with all other variables, including personal responses to the same questions (Fig 2). The plot shows 587 pairwise significant (p < 0.05) variable correlations, of which 60 remain significant after Bonferroni correction. The pattern is dominated by a strong diagonal series consistent with the false consensus effect [29,30] as each respondent believed their own viewpoint was shared by the majority for a mean of 12.2 ± 0.2 out of 20 questions asked of them (standard deviation 3.4).

**Fig 3:**
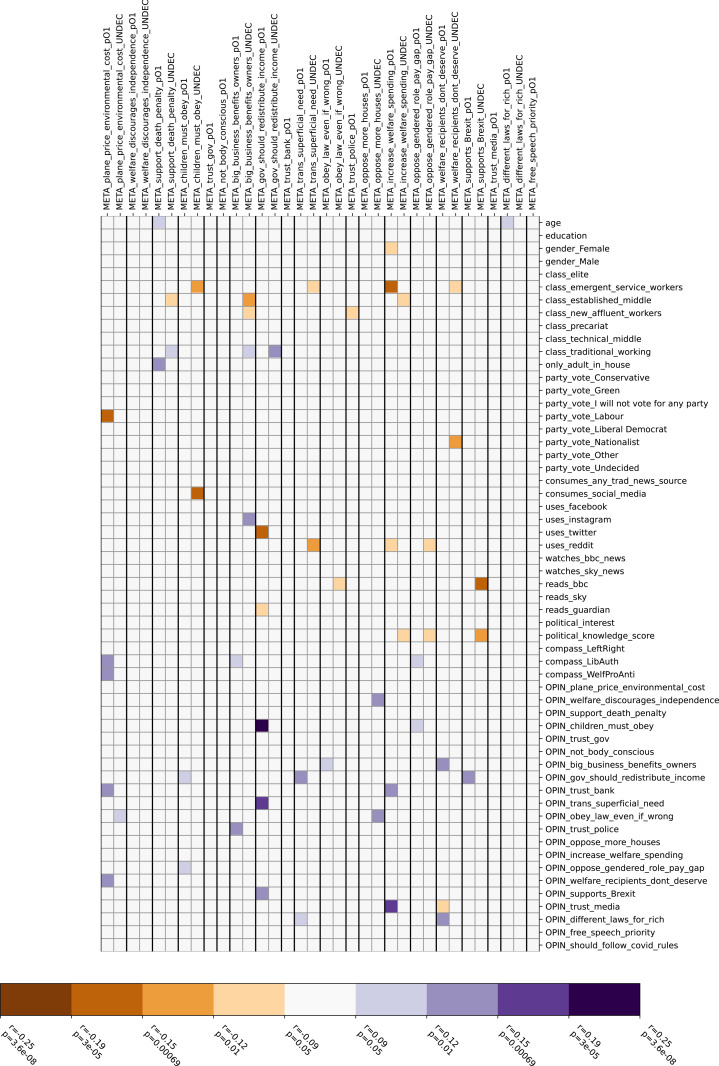
Correlations that cannot be explained by, but do not contradict, the false consensus effect.

**Fig 4:**
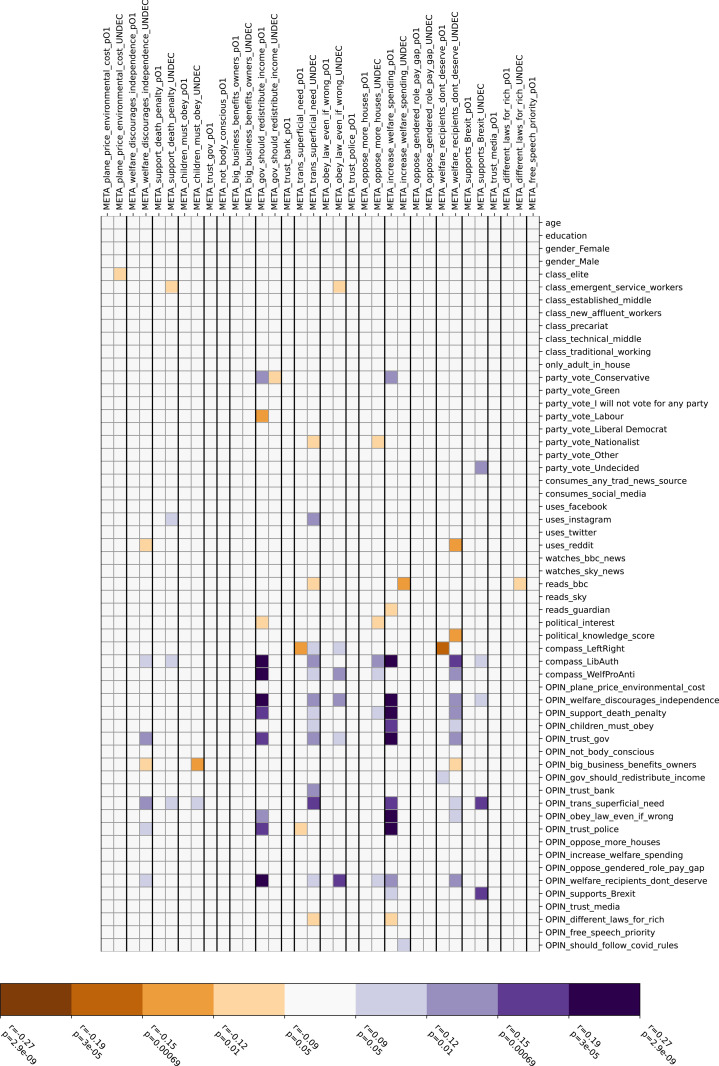
Correlations that contradict the false consensus effect.

Figs 3 and 4 show similar correlation matrices, albeit filtering out correlations which could in principle be explained by the false consensus effect. Fig 3 shows correlations unexplained by, but not contradicting false consensus; 55 of the original 587 pairwise significant correlations fall into this category, but only one of these remains significant after Bonferroni correction. Fig 4 shows correlations which contradict the false consensus effect; 89 of the original pairwise correlations (9 after Bonferroni correction) fall into this category. These arise mostly from six specific questions, two with strong correlations (gov_should_redistribute_income, increase_welfare_spending, p < 0.05 after Bonferroni correction) and four with weaker evidence (obey_law_even_if_wrong, oppose_more_houses, trans_superficial_need, welfare_recipients_dont_deserve, p < 0.05 only before Bonferroni correction). Responses to the remaining 14 questions conform to the false consensus hypothesis.

Results from our attempts to cluster response data in an attempt to detect the effect of discrete echo chambers on meta-opinions, found little clustering tendency, with the maximum silhouette score across all clustering runs being 0.09 (for k-means fitting of 2 clusters) out of a possible maximum silhouette score of 1.

When re-tested on their two worst scoring questions, participants improved their scores by a mean of 2.2 bits per question (±0.06, n = 952). 76% of participants rated the quiz as either “quite” or “very” interesting, 22% as “a little interesting” and 2% as “not at all interesting”.

## Discussion

The finding that knowledge of others’ opinions is consistent for each respondent, that is, someone scoring highly on one question is more likely to score a high average on other questions, shows that the information theoretic scoring technique is self-consistent.

Our finding of small changes in repeated BSAS questions show that if change of public opinion over the elapsed 3 years between original survey (BSAS), and our own meta-opinion survey, is affecting respondent performance, its explanatory power would be limited: the average respondent error is over three times the difference accounted for by this public opinion change. This is corroborated by BSAS’s own summary at the time, “there is little sign that the pandemic has instigated any marked shift of attitudes in a new direction” [55], and consistent with literature on attitude changes considering longer periods of 18–54 years [11].

Again it should be noted that in interpreting the remainder of these findings, although the sample used includes all major political parties, levels of education and responses to our opinion questions, it is self-selected rather than statistically representative.

The finding that particular social media platforms appear to have an effect on the correctness of meta-opinions, warrants brief discussion. The negative effect of Instagram possibly reflects the platform’s focus on photo sharing rather than public conversations. The positive effect for Reddit is consistent with the findings of [4] and potentially reflects the diversity of Reddit sub-forums.

The strong false consensus effect observed in all correlation matrices is consistent with the literature on this topic [11,29,30]. For correlations unexplained by false consensus, we cannot discern any general explanation though we note that only one of these correlations remains significant after Bonferroni correction, suggesting that at least some of these correlations may be spurious. Some of the correlations which contradict false consensus also become insignificant after Bonferroni correction, although a potential interpretation can be offered notwithstanding: namely that on certain questions, people may expect others to remain undecided although they themselves have picked a side (obey_law_even_if_wrong, oppose_more_houses, trans_superficial_need, welfare_recipients_dont_deserve).

The correlations which contradict false consensus and remain significant after Bonferroni correction, however, are not so easily dismissed. These relate in particular to welfare questions (gov_should_redistribute_income, increase_welfare_spending) showing that on this particular topic, respondents are more aware of opposing viewpoints. The literature on false consensus acknowledges that cultural and situational variables can mediate [18], so it is not surprising to find an exception concentrated on a specific topic.

The key null result is the lack of clustering apparent in the meta-opinion data in isolation. For clarity it is worth emphasizing that the clustering analysis in this paper is applied to meta-opinions themselves, and not social network data as with existing literature. It tests whether the respondents can be divided into well-defined groups based on their meta-opinions alone: for example given a set of possible opinions {A, B, C, …}, can you roughly describe the respondents as a small number of groups, where one group believes the majority think ABCDE, another group believes the majority think FGHIJ, and so on. To cluster in this manner it is not necessary that a group agrees unanimously on what others think; for example in the ABCDE group some may believe ABCD while others believe BDE or even - in moderation - include beliefs from other groups, for example BDEJ. What would matter overall is the extent to which this group is separable from other, obviously different groups such as FGHIJ. What we have found, however is that within our data such groups cannot be well-defined; i.e., there are enough people combining characteristics of multiple groups, with diverse beliefs about majority opinion such as ACEGI or BDFHJ, that it does not make any sense to group respondents in this manner.

This is a surprising finding for meta-opinions in particular, given that both our own data, and the recent work of others [11,12] has shown significant false consensus effects correlating to demographics, media consumption and political opinion variables, in a context where much related research supports the existence of echo chambers. One might therefore expect to find clusters in the data, each representing a an echo chamber shared by a defined political group. The lack of clustering is, however, consistent with our finding that the false consensus effect also applies when people are undecided on an issue: in a scenario dominated by echo chambers we would expect even undecided people to perceive others as having made up their minds, whereas in fact our data show that this is not the case.

The contrast between this finding and those in the literature which found strong evidence for echo chambers, can be explained by differences in operationalizing the echo chamber concept. [18] studies public statements and endorsement, rather than private perceptions of opinion distribution, which – considering spiral of silence effects - we would expect to be different. Maestroianni and Dana [11] found that more liberal participants in the US were better at estimating long-term attitude change, while Boutyline and Willer [6] measured online interactions in the US, finding (as with [22,56]) that conservatives tend to greater homophily. All of these align with our own UK finding in which conservatives have less knowledge of meta-opinions. Drews et al. [13], although not an echo chamber study, finds strong false consensus bias among those opposed to carbon taxation in Spain, and the opposite among those in favour of it. From analysis of our own question on this topic (“The price of a plane ticket should reflect the environmental damage that flying causes, even if this makes air travel much more expensive”) we also find that those in favour of this policy showed greater awareness of opposing opinions on this particular question (strong correlation of this opinion with its SCORE variable in Fig 2), but not greater awareness of other opinions when considering all topics together (Fig 1).

Other studies finding homophilic interactions cover US climate policy networks [7] and social media [17]. In our own findings it would appear that such homophily of interactions does not translate into clustering of meta-opinions, presumably because people can be well aware of opposing views even though they prefer to interact with those of like minds. Established findings of significant cross-cutting interactions on US Twitter [8], cross-cutting US media consumption [24,26], and weakness of clustering in Australian Twitter [23] would all appear to corroborate this.

Interestingly, our findings contrast with Boutyline and Willer [6] when it comes to political extremes on both sides, for whom the cited study finds stronger homophily, but we find to have greater awareness. We speculate that political extremes perhaps represent a conscious choice of position based on greater involvement in politics and hence are associated with greater awareness of opposing views, despite preference for associating with like-minded people. Dubois and Blank [25], studying the UK as we do here, finds cross-cutting media consumption to be stronger for those of stronger political interests, based on self-reported data on how often respondents read material they disagree with.

Levy [27] tracks the effects of subscribing US subjects’ Facebook feeds to different news sources, and measuring resulting changes to exposure, visits, posts and attitude change. The primary measure of interest is affective polarization, defined as negative attitudes towards the opposing political party, which is found to decrease with exposure to opposing news sources, even though the respondents’ own opinions (issue polarization) remained unchanged (this categorization of two types of polarization is based on [57]). In the context of the current study, the relevant point is that how people *feel* about political opponents is distinct from whether they *agree* with them. Our own work has asked respondents for their own opinions, hence measuring issue polarization and finding no echo chamber effects, but it is entirely possible that echo-chamber-like discourse exists in the domain of feelings (affective polarization).

## Conclusions

This paper presents an exploratory analysis of the space of political meta-opinions, based on a gamified survey deploying a new method for information-theoretic scoring of meta-opinion accuracy. We find that different levels of awareness of other opinions are associated with political interest, knowledge and alignment, and own opinions on a range of topics. We also find associations between own opinions and meta-opinions, consistent with a false consensus effect whereby people are biased towards believing that others think like themselves.

Terren and Borge-Bravo’s systematic review [14] concluded that articles finding clear evidence of echo chambers were largely based on digital trace data (e.g., of interactions on social media), while those finding no evidence were all based on self-reported data. Furthermore this review suggested that digital trace data may overestimate the presence of echo chambers, while self-reported data may underestimate the same, and concluded that it would be valuable to develop more hybrid approaches. Here we have proposed an intermediate approach: although based on self-reported data, the meta-opinion parts of our survey/game are likely less susceptible to bias caused by respondents aiming to give approval-seeking answers, as the ‘right’ or ‘approved’ answer is unknown to the respondents (although this does not apply to own-opinion or media consumption questions). Our technique also circumvents some of the challenges with existing approaches identified by Mahmoudi et al. [15], in particular, dispensing with (1) the assumption that if echo chambers are present, there will be no more than two distinct groups; and (2) the assumption that polarization is a prerequisite for an echo chamber. As such, it has been useful for the measurement of biases in awareness across a range of topics, and how those biases vary with political alignment.

Structuring the meta-opinion survey as a game, which was enabled by the information-theoretic scoring approach, proved to be a useful strategy not only for collecting the data, but also in educating participants on the balance of public opinion. The improved scores during unannounced retesting demonstrate that participants had paid attention to their own results page – even though they were not required to read details of their results. The positive feedback received from participants was further supported by free text comments we collected, which expressed mostly interest, enjoyment or surprise at the results. Further work could explore the use of similar games in political and social education.

Although previous research on measuring echo chambers via various data sources has yielded both strong and weak results, in the current case of meta-opinion data, we were surprised by the lack of meta-opinion clusters representing echo chambers.

We suggest that the study is limited by the fact that, while meta-opinions are strongly linked to echo chambers, the absence of errors in meta-opinions themselves doesn’t itself indicate absence of an echo chamber. An echo chamber is likely to have differing effects on (1) awareness of others opinions (as measured here), (2) opinions of others opinions, (3) emotional reactions to others opinions [27], (4) opinions publicly shared within and outside the echo chamber, and (5) opinions held privately. It is plausible that echo chambers may exhibit biases in any of these, without biased awareness - as mere awareness of a diverse range of opinions is not the same thing as public or private agreement with, or respect for, those opinions. The factors influencing meta-opinion accuracy can capture dynamics surrounding an echo chamber, while the meta-opinions themselves are better described by false consensus rather than echo chamber effects. Biases in the meta-opinions we observed appear to be unique to each individual, rather than defined by population-level groups.

Future work could overcome this limitation by linking measures of echo chamber structure and reinforcement to the semantic measures of opinion and meta-opinion, by including survey questions on interactions, agreements and disagreements with peers, or incorporating social media data. Emotional responses to opinions held by others may be particularly suited to a similar analysis as conducted here. Following review and classification of the range of emotional responses, questions such as the following could be asked:

*Some people believe XXX. What proportion of the UK do you think*

1. *Would agree?*
2. *Would disagree (without particular emotion)?*
3. *Would feel angry or threatened by this belief?*

*Considering the people who believe XXX,* what *proportion do you think would be comfortable sharing this opinion?*

For both of these questions, the degree to which respondents can correctly identify proportions of the UK in each category – as quantified by the framework used in the current study – may be more revealing of echo chambers.

A final limitation is the sample, which – although including a wide representation of demographics and opinions – is self-selected rather than statistically representative, and also cross-sectional. Further work could consider a representative sample to strengthen the evidence on this topic, and also sampling at multiple points in time to capture the dynamic aspects of echo chamber formation and dissolution.

## Supporting information

S1 File Survey questions. (DOCX)

S2 File Definition of variables and descriptive statistics. (DOCX)

S3 File Code and data. (ZIP)
