# Supplementary material for: Biased perceptions of public opinion don’t define echo chambers but reveal systematic differences in political awareness
Source: PLoS One. 2025 Jun 4;20(6):e0324507. doi: 10.1371/journal.pone.0324507 (PMC12178016; doi:10.1371/journal.pone.0324507)
Supplement: S1 File — (DOCX) [file pone.0324507.s001.docx]

| How much interest would you say you have in politics? | |
| --- | --- |
|  | ... a great deal, |
|  | quite a lot, |
|  | some, |
|  | not very much, |
|  | or, none at all? |
|  |  |
| The following questions are designed to assess your level of knowledge of UK politics. Please do not google for answers - you will not be penalized in any way if you get them wrong! | |
| What proportion of seats is required to pass a Bill in the House of Commons? | |
|  | Just over half (326 of 650 seats) |
|  | 60% (390 of 650 seats) |
|  | 66% (429 of 650 seats) |
|  | 76% (494 of 650 seats) |
|  | I don't know |
|  |  |
| What is the leading source of electricity in the United Kingdom? | |
|  | Renewables (wind, solar, hydro) |
|  | Coal |
|  | Natural Gas |
|  | Nuclear |
|  | I don't know |
|  |  |
| Which party currently has the most seats in the House of Commons? | |
|  | Conservative Party |
|  | Labour Party |
|  | Liberal Democratic Party |
|  | Scottish National Party |
|  | I don't know |
|  |  |
| Do you read any morning paper 3 or more times per week? [yes/no. if yes, free text question - which one?] | |
|  |  |
| Do you read any news website 3 or more times per week? [yes/no. if yes, free text question - which one?] | |
|  |  |
| Do you watch the news on television (or an online streaming service such as iPlayer) 3 or more times per week? [yes/no. if yes, free text question - which channel?] | |
|  |  |
| Do you read any social media site (e.g. Facebook, Twitter) 3 or more times per week? [yes/no. if yes, free text question - which ones?] | |
|  |  |
| Which political party would you be most likely to vote for at the next election? | |
|  | Conservative |
|  | Green |
|  | Labour |
|  | Liberal Democrat |
|  | Scottish National |
|  | Plaid Cymru |
|  | Other |
|  | Undecided |
|  | I will not vote for any party |
|  |  |
| Quiz: How well do you know the British public?  How To Play  Firstly, select your personal answer to the question. Then, try to guess the percentage of what the general public thought using the sliders. Click the 'Confirm' button to save your answer and advance to the next question. Good luck! | |
|  |  |
| Do you tend to trust or tend not to trust the media? | |
|  | Trust |
|  | Distrust |
|  |  |
| Do you tend to trust or tend not to trust the police? | |
|  | Trust |
|  | Distrust |
|  |  |
| Do you tend to trust or tend not to trust high street banks and financial institutions? | |
|  | Trust |
|  | Distrust |
|  |  |
| Do you tend to trust or tend not to trust the Government? | |
|  | Trust |
|  | Distrust |
|  |  |
| Which of these statements comes closest to your view about extremism vs free speech in universities and colleges? | |
|  | Universities have responsibility to protect free speech on campus |
|  | Universities have responsibility to protect students from hearing extremist views |
|  |  |
| Government should redistribute income from the better-off to less well-off | |
|  | Agree |
|  | Neither agree nor disagree |
|  | Disagree |
|  |  |
| Big business benefits owners at the expense of workers | |
|  | Agree |
|  | Neither agree nor disagree |
|  | Disagree |
|  |  |
| There is one law for the rich and one for the poor | |
|  | Agree |
|  | Neither agree nor disagree |
|  | Disagree |
|  |  |
| For some crimes, the death penalty is the most appropriate sentence | |
|  | Agree |
|  | Neither agree nor disagree |
|  | Disagree |
|  |  |
| Schools should teach children to obey authority | |
|  | Agree |
|  | Neither agree nor disagree |
|  | Disagree |
|  |  |
| The law should always be obeyed, even if a particular law is wrong | |
|  | Agree |
|  | Neither agree nor disagree |
|  | Disagree |
|  |  |
| The government should spend more money on welfare benefits for the poor | |
|  | Agree |
|  | Neither agree nor disagree |
|  | Disagree |
|  |  |
| Many people who get social security don’t really deserve any help | |
|  | Agree |
|  | Neither agree nor disagree |
|  | Disagree |
|  |  |
| If welfare benefits weren’t so generous, people would learn to stand on their own two feet | |
|  | Agree |
|  | Neither agree nor disagree |
|  | Disagree |
|  |  |
| Would you support or oppose more homes being built in your local area? | |
|  | Oppose |
|  | Neither support nor oppose |
|  | Support |
|  |  |
| The price of a plane ticket should reflect the environmental damage that flying causes, even if this makes air travel much more expensive | |
|  | Agree |
|  | Neither agree nor disagree |
|  | Disagree |
|  |  |
| Does how you look affect how you feel about yourself? | |
|  | How I look doesn`t really affect how I feel about myself |
|  | I feel much better about myself when I think I look good |
|  |  |
| How right or wrong it is for men to be paid more on average than women in a supermarket where most of the check out workers are women and most of the managers are men? | |
|  | Wrong |
|  | Neither right nor wrong |
|  | Right |
|  |  |
| Do you agree or disagree that most people who are transgender have gone through this process because of a very superficial and temporary need? | |
|  | Agree |
|  | Neither agree nor disagree |
|  | Disagree |
|  |  |
| Considering Brexit, do you now think of yourself as a Remainer or a Leaver? | |
|  | Leaver |
|  | I do not think of myself as either remainer or leaver |
|  | Remainer |
|  |  |
| Further questions: | |
|  |  |
| Thinking of the restrictions imposed during coronavirus (COVID-19) lockdown, do you think | |
|  | It’s important to follow the rules completely |
|  | It’s important to follow the rules for the most part |
|  | It doesn’t matter whether or not the rules are followed |
|  |  |
| Please take the Great British Class Survey available at (link) | |
| What is your own result? | |
|  | Elite |
|  | Established Middle Class |
|  | Technical Middle Class |
|  | New Affluent Workers |
|  | Traditional Working Class |
|  | Emergent Service Workers |
|  | Precariat |
|  |  |
| Your gender | |
|  | Female |
|  | Male |
|  | Other |
|  |  |
| Your age |  |
|  | 18-25 |
|  | 26-35 |
|  | 36-45 |
|  | 46-55 |
|  | 56-65 |
|  | >66 |
|  |  |
| What is your highest educational qualification? | |
|  | Degree level or higher |
|  | Higher education below degree level |
|  | A level or equivalent |
|  | GCSE, O level or equivalent |
|  | No qualification |
|  |  |
| Besides yourself are there any other adults in your household? | |
|  | Yes |
|  | No |
