# Supplementary material for: Biased perceptions of public opinion don’t define echo chambers but reveal systematic differences in political awareness
Source: PLoS One. 2025 Jun 4;20(6):e0324507. doi: 10.1371/journal.pone.0324507 (PMC12178016; doi:10.1371/journal.pone.0324507)
Supplement: S2 File — (DOCX) [file pone.0324507.s002.docx]

# Definition of variables and descriptive statistics

Notes:

1. Age was measured as a categorical: [18-25, 26-35, 36-45, 46-55, 56-65, 66+]. Each category is encoded at its midpoint, except 66+ which is encoded as 66.
2. Education is encoded as an ordinal variable with categories: [No qualification; GCSE, O level or equivalent; A level or equivalent; Higher education below degree level; Degree level or higher]
3. Self reported political interest is encoded as an ordinal variable with 5 categories [“None at all”…“A great deal”]
4. Political knowledge score is out of 3, normalized to 1
5. Each political compass variable is aggregated from three relevant opinion variables. LeftRight2, LibAuth2, WelfProAnti2, OPIN_takes_sides_Brexit are derived from squaring the respective variables when scaled to a range of (-1,1) to indicate extreme viewpoints on either side of each scale.
6. We measured opinion variable OPIN_should_follow_covid_rules without metavariable as there was no BSAS data for comparison.
7. Social class categories derived from Savage, M., Devine, F., Cunningham, N., Taylor, M., Li, Y., Hjellbrekke, J., Le Roux, B., Friedman, S., Miles, A., 2013. A New Model of Social Class? Findings from the BBC’s Great British Class Survey Experiment. Sociology 47, 219–250. https://doi.org/10.1177/0038038513481128

| Category notes | Variable | mean | std | min | max |
| --- | --- | --- | --- | --- | --- |
| Demographics  All variables are self-reported. | age (a) | 32 | 10 | 21 | 66 |
|  | education (b) | 0.79 | 0.27 | 0. | 1. |
|  | gender_Female | 0.30 | 0.46 | 0. | 1. |
|  | gender_Male | 0.69 | 0.46 | 0. | 1. |
|  | gender_Other | 0.004 | 0.06 | 0. | 1. |
|  | class_elite | 0.05 | 0.23 | 0. | 1. |
|  | class_emergent_service_workers | 0.28 | 0.45 | 0. | 1. |
|  | class_established_middle | 0.16 | 0.36 | 0. | 1. |
|  | class_new_affluent_workers | 0.05 | 0.22 | 0. | 1. |
|  | class_precariat | 0.05 | 0.22 | 0. | 1. |
|  | class_technical_middle | 0.13 | 0.33 | 0. | 1. |
|  | class_traditional_working | 0.11 | 0.31 | 0. | 1. |
|  | only_adult_in_house | 0.14 | 0.35 | 0. | 1. |
| Media consumption | consumes_any_trad_news_source | 0.87 | 0.33 | 0. | 1. |
|  | consumes_social_media | 0.83 | 0.37 | 0. | 1. |
|  | uses_facebook | 0.41 | 0.49 | 0. | 1. |
|  | uses_instagram | 0.07 | 0.26 | 0. | 1. |
|  | uses_twitter | 0.24 | 0.43 | 0. | 1. |
|  | uses_reddit | 0.05 | 0.23 | 0. | 1. |
|  | watches_bbc_news | 0.35 | 0.48 | 0. | 1. |
|  | watches_sky_news | 0.06 | 0.24 | 0. | 1. |
|  | reads_bbc | 0.42 | 0.49 | 0. | 1. |
|  | reads_sky | 0.03 | 0.17 | 0. | 1. |
|  | reads_guardian | 0.06 | 0.23 | 0. | 1. |
| Political | political_interest (c) | 0.56 | 0.23 | 0. | 1. |
|  | political_knowledge_score (d) | 0.64 | 0.27 | 0. | 1. |
|  | party_vote_Conservative | 0.20 | 0.40 | 0. | 1. |
|  | party_vote_Green | 0.08 | 0.27 | 0. | 1. |
|  | party_vote_I will not vote for any party | 0.06 | 0.24 | 0. | 1. |
|  | party_vote_Labour | 0.35 | 0.48 | 0. | 1. |
|  | party_vote_Liberal Democrat | 0.09 | 0.28 | 0. | 1. |
|  | party_vote_Nationalist | 0.05 | 0.22 | 0. | 1. |
|  | party_vote_Other | 0.04 | 0.19 | 0. | 1. |
|  | party_vote_Undecided | 0.13 | 0.34 | 0. | 1. |
|  | compass_LeftRight (e) | 0.24 | 0.26 | 0. | 1. |
|  | compass_LibAuth (e) | 0.49 | 0.31 | 0. | 1. |
|  | compass_WelfProAnti (e) | 0.35 | 0.32 | 0. | 1. |
|  | compass_LeftRight2 (e) | 0.54 | 0.40 | 0. | 1. |
|  | compass_LibAuth2 (e) | 0.38 | 0.38 | 0. | 1. |
|  | compass_WelfProAnti2 (e) | 0.50 | 0.41 | 0. | 1. |
| Quiz results  SCORE_MEAN denotes mean bits of information correctly guessed per question compared to mean random response (negative figures indicate worse than random guess)  OPIN_ denotes respondent opinion for each question, encoded as ordinal  META_ denotes meta-opinions:   - Suffix _UNDEC denotes proportion of population respondent believes would be neutral/undecided (corresponding to ordinal encoding of 0.5 in OPIN variable) - Suffix _pO1 denotes proportion of population respondent believes chose option 1 (corresponding to ordinal encoding of 1 in OPIN variable) - Suffix _SCORE denotes bits of information correctly guessed by respondent on each question | SCORE_MEAN | 0.60 | 0.51 | -0.98 | 1.77 |
|  | OPIN_plane_price_environmental_cost | 0.55 | 0.44 | 0. | 1. |
|  | META_plane_price_environmental_cost_pO1 | 0.44 | 0.27 | 0. | 1. |
|  | META_plane_price_environmental_cost_UNDEC | 0.24 | 0.15 | 0. | 0.8 |
|  | META_plane_price_environmental_cost_SCORE | 0.23 | 1.46 | -1.43 | 6.15 |
|  | OPIN_welfare_discourages_independence | 0.42 | 0.44 | 0. | 1. |
|  | META_welfare_discourages_independence_pO1 | 0.60 | 0.24 | 0. | 1. |
|  | META_welfare_discourages_independence_UNDEC | 0.23 | 0.14 | 0. | 0.72 |
|  | META_welfare_discourages_independence_SCORE | 0.83 | 1.66 | -1.43 | 7.31 |
|  | OPIN_support_death_penalty | 0.46 | 0.46 | 0. | 1. |
|  | META_support_death_penalty_pO1 | 0.53 | 0.27 | 0. | 1. |
|  | META_support_death_penalty_UNDEC | 0.23 | 0.15 | 0. | 0.89 |
|  | META_support_death_penalty_SCORE | 0.58 | 1.55 | -1.41 | 7.31 |
|  | OPIN_children_must_obey | 0.56 | 0.42 | 0. | 1. |
|  | META_children_must_obey_pO1 | 0.64 | 0.26 | 0. | 1. |
|  | META_children_must_obey_UNDEC | 0.24 | 0.15 | 0. | 0.99 |
|  | META_children_must_obey_SCORE | 0.84 | 1.48 | -1.43 | 8.9 |
|  | OPIN_trust_gov | 0.39 | 0.49 | 0. | 1. |
|  | META_trust_gov_pO1 | 0.47 | 0.21 | 0. | 0.97 |
|  | META_trust_gov_SCORE | 0.53 | 1.36 | -1.35 | 5.26 |
|  | OPIN_not_body_conscious | 0.28 | 0.45 | 0. | 1. |
|  | META_not_body_conscious_pO1 | 0.39 | 0.25 | 0. | 1. |
|  | META_not_body_conscious_SCORE | 0.30 | 1.31 | -1.4 | 5.26 |
|  | OPIN_big_business_benefits_owners | 0.86 | 0.30 | 0. | 1. |
|  | META_big_business_benefits_owners_pO1 | 0.73 | 0.21 | 0. | 1. |
|  | META_big_business_benefits_owners_UNDEC | 0.22 | 0.15 | 0. | 1. |
|  | META_big_business_benefits_owners_SCORE | 1.29 | 1.60 | -1.41 | 8.9 |
|  | OPIN_gov_should_redistribute_income | 0.70 | 0.38 | 0. | 1. |
|  | META_gov_should_redistribute_income_pO1 | 0.60 | 0.23 | 0. | 1. |
|  | META_gov_should_redistribute_income_UNDEC | 0.28 | 0.16 | 0. | 1. |
|  | META_gov_should_redistribute_income_SCORE | 0.84 | 1.46 | -1.43 | 7.58 |
|  | OPIN_trust_bank | 0.56 | 0.50 | 0. | 1. |
|  | META_trust_bank_pO1 | 0.54 | 0.25 | 0. | 0.99 |
|  | META_trust_bank_SCORE | 0.36 | 1.40 | -1.4 | 5.26 |
|  | OPIN_should_follow_covid_rules | 0.77 | 0.29 | 0. | 1. |
|  | OPIN_trans_superficial_need | 0.32 | 0.39 | 0. | 1. |
|  | META_trans_superficial_need_pO1 | 0.49 | 0.27 | 0. | 1. |
|  | META_trans_superficial_need_UNDEC | 0.28 | 0.18 | 0. | 1. |
|  | META_trans_superficial_need_SCORE | 0.28 | 1.48 | -1.43 | 8.09 |
|  | OPIN_obey_law_even_if_wrong | 0.44 | 0.43 | 0. | 1. |
|  | META_obey_law_even_if_wrong_pO1 | 0.57 | 0.25 | 0. | 1. |
|  | META_obey_law_even_if_wrong_UNDEC | 0.27 | 0.16 | 0. | 1. |
|  | META_obey_law_even_if_wrong_SCORE | 0.82 | 1.98 | -1.43 | 8.58 |
|  | OPIN_trust_police | 0.68 | 0.47 | 0. | 1. |
|  | META_trust_police_pO1 | 0.63 | 0.20 | 0. | 1. |
|  | META_trust_police_SCORE | 0.60 | 1.32 | -1.4 | 5.26 |
|  | OPIN_oppose_more_houses | 0.36 | 0.42 | 0. | 1. |
|  | META_oppose_more_houses_pO1 | 0.56 | 0.27 | 0. | 1. |
|  | META_oppose_more_houses_UNDEC | 0.24 | 0.16 | 0. | 1. |
|  | META_oppose_more_houses_SCORE | 0.17 | 1.50 | -1.43 | 9.31 |
|  | OPIN_increase_welfare_spending | 0.73 | 0.39 | 0. | 1. |
|  | META_increase_welfare_spending_pO1 | 0.59 | 0.25 | 0. | 1. |
|  | META_increase_welfare_spending_UNDEC | 0.22 | 0.15 | 0. | 1. |
|  | META_increase_welfare_spending_SCORE | 0.68 | 1.73 | -1.43 | 6.31 |
|  | OPIN_oppose_gendered_role_pay_gap | 0.61 | 0.42 | 0. | 1. |
|  | META_oppose_gendered_role_pay_gap_pO1 | 0.62 | 0.27 | 0. | 1. |
|  | META_oppose_gendered_role_pay_gap_UNDEC | 0.23 | 0.18 | 0. | 1. |
|  | META_oppose_gendered_role_pay_gap_SCORE | 0.37 | 1.46 | -1.43 | 7.58 |
|  | OPIN_welfare_recipients_dont_deserve | 0.36 | 0.41 | 0. | 1. |
|  | META_welfare_recipients_dont_deserve_pO1 | 0.54 | 0.24 | 0. | 1. |
|  | META_welfare_recipients_dont_deserve_UNDEC | 0.25 | 0.16 | 0. | 1. |
|  | META_welfare_recipients_dont_deserve_SCORE | 0.27 | 1.47 | -1.43 | 4.77 |
|  | OPIN_supports_Brexit | 0.30 | 0.40 | 0. | 1. |
|  | META_supports_Brexit_pO1 | 0.51 | 0.21 | 0. | 1. |
|  | META_supports_Brexit_UNDEC | 0.18 | 0.15 | 0. | 1. |
|  | META_supports_Brexit_SCORE | 1.10 | 1.54 | -1.43 | 8.09 |
|  | OPIN_trust_media | 0.38 | 0.49 | 0. | 1. |
|  | META_trust_media_pO1 | 0.47 | 0.21 | 0. | 0.96 |
|  | META_trust_media_SCORE | 0.23 | 1.41 | -1.31 | 5.29 |
|  | OPIN_different_laws_for_rich | 0.73 | 0.40 | 0. | 1. |
|  | META_different_laws_for_rich_pO1 | 0.68 | 0.24 | 0. | 1. |
|  | META_different_laws_for_rich_UNDEC | 0.23 | 0.15 | 0. | 0.84 |
|  | META_different_laws_for_rich_SCORE | 1.12 | 1.62 | -1.43 | 10.9 |
|  | OPIN_free_speech_priority | 0.73 | 0.45 | 0. | 1. |
|  | META_free_speech_priority_pO1 | 0.61 | 0.21 | 0. | 1. |
|  | META_free_speech_priority_SCORE | 0.46 | 1.27 | -1.4 | 5.26 |
| Extra opinion variables | OPIN_should_follow_covid_rules (f) | 0.77 | 0.29 | 0. | 1. |
|  | OPIN_takes_sides_Brexit (e) | 0.79 | 0.40 | 0. | 1. |
